# A Perplexing Case of Bladder Mass Biopsy-Proven Neurosarcoidosis

**DOI:** 10.7759/cureus.40865

**Published:** 2023-06-23

**Authors:** Zainab Hanif, Keysha N Gonzalez Ramos, Pouyan Razminia, Eboselum Aigbe, Pegah Ghafourian

**Affiliations:** 1 Internal Medicine, HCA Florida Orange Park Hospital, Orange Park, USA

**Keywords:** neoplasm, mimicking, nodular leptomeningeal enhancement, bladder mass, neurosarcoidosis

## Abstract

Sarcoidosis is a multi-organ systemic disease that presents with several clinical manifestations, and patients can develop neurologic complications. Neurosarcoidosis may be life-threatening; therefore, early recognition and treatment are key. Here, we present a case of a 55-year-old African American male who presented with a complaint of dizziness and left-sided weakness; he ultimately received a diagnosis of neurosarcoidosis after elaborate radiographic investigations and bladder mass biopsy. Neurosarcoidosis remains a diagnostic dilemma as it can clinically and radiographically mimic multiple conditions including multiple sclerosis, central nervous system lymphoma, multiple myeloma, and progressive multifocal leukoencephalopathy.

## Introduction

Neurosarcoidosis is a challenging medical condition where up to 70% of patients present with neurological manifestations rather than already having a known systemic diagnosis [1]. It is difficult to diagnose as the disease can present in many different ways [2]. The diagnosis is usually one of exclusion as it often masquerades as other disorders, at times creating a lengthy differential and complicated diagnosis [3]. There is no cure, and most patients require long-term treatment with corticosteroids.

This case was previously presented as a poster at the Florida Society of Rheumatology Annual Fellow Poster Presentation on July 09, 2022.

## Case presentation

**Figure 1:**
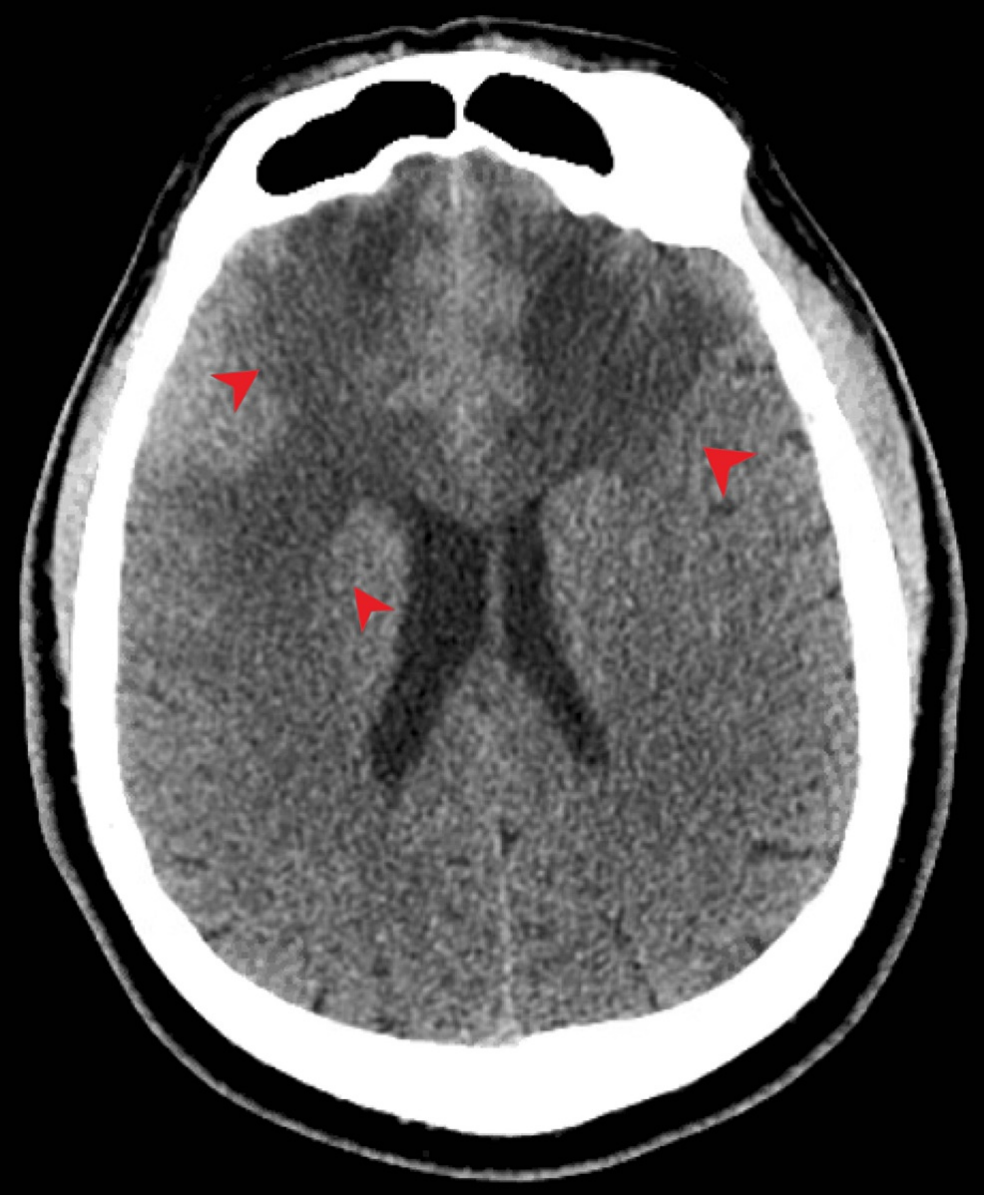
CT scan of the brain (axial view), on arrival, demonstrating large areas of white matter hypodensities in the bifrontal lobes as well as small areas of cortical hypodensities in the right anteroinferior frontal lobe (arrows)

A 55-year-old African American male, with a medical history of substance use, cerebrovascular accident, hepatitis B, recently diagnosed with right lower extremity deep venous thrombosis on anticoagulation presented to the hospital with complaints of dizziness, blurry vision and left-sided weakness of one-day duration. Of note, the patient had had multiple emergency department visits within the past month for similar left-sided leg pain and falls. He had been treated for cellulitis and pain. This time computed tomography (CT) of the brain was ordered and revealed large areas of white matter hypodensities in bifrontal lobes as well as small areas of cortical hypodensities in the right anterioinferior frontal lobe (Figure 1).

This was initially concerning for acute infarct versus subacute post-traumatic changes. Other possible differentials were reversible posterior leukoencephalopathy or neoplastic process given the local mass effect. Neurological examination was non-focal and cranial nerves II-XII were intact. A painful range of motion was noted in the lower extremity; however, muscle strength was noted to be 5/5. Additional findings on the physical examination included bilateral lower extremity edema left more than right, oral thrush and right axillary lymphadenopathy. The stroke protocol was initiated, and the patient was admitted to the hospital. Neurology and Hematology Oncology services were consulted for further evaluation.

**Figure 2:**
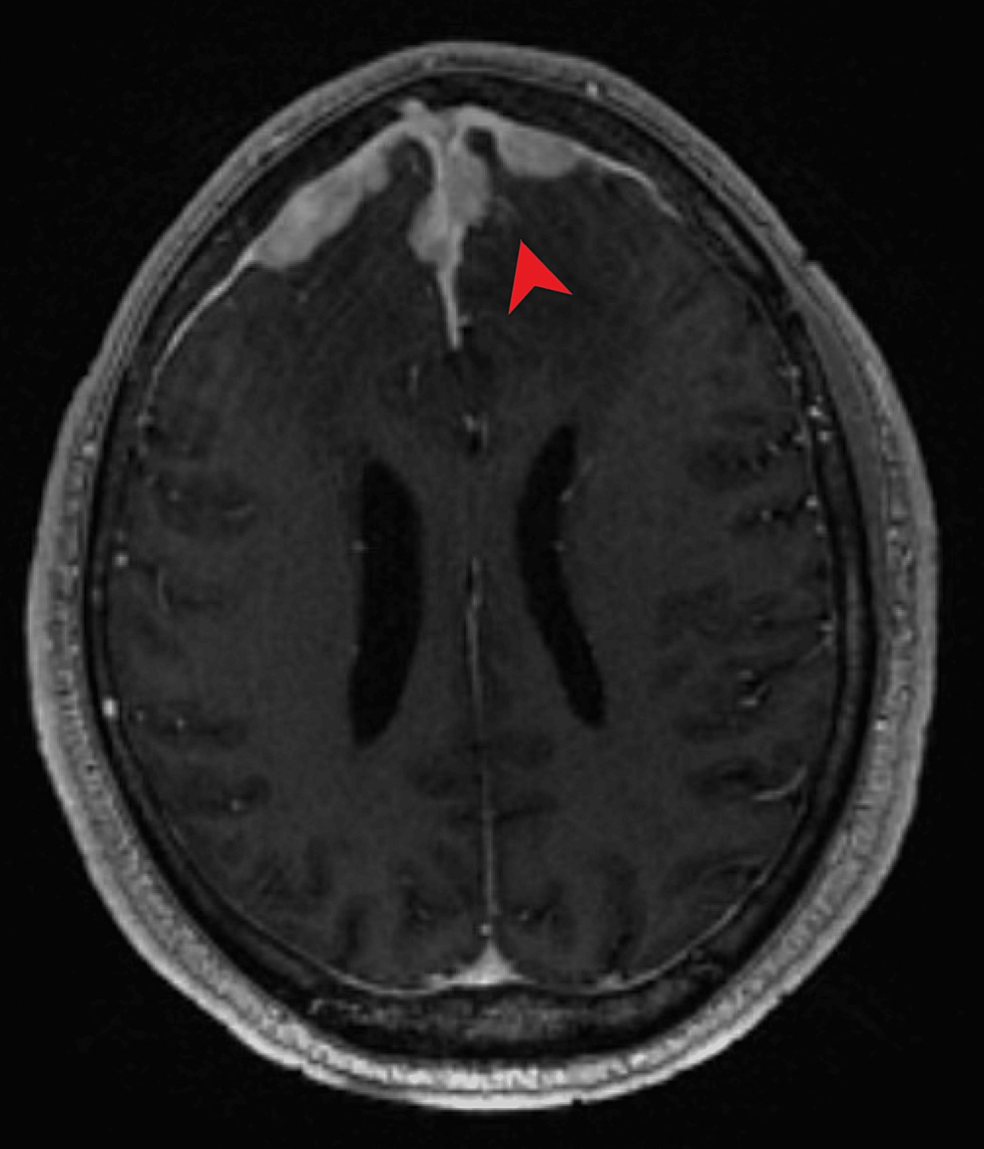
Axial T1-weighted MRI of the brain with contrast showing extensive enhancing dural nodularity along bilateral frontal convexities and anterior falx (red arrow)

**Figure 3:**
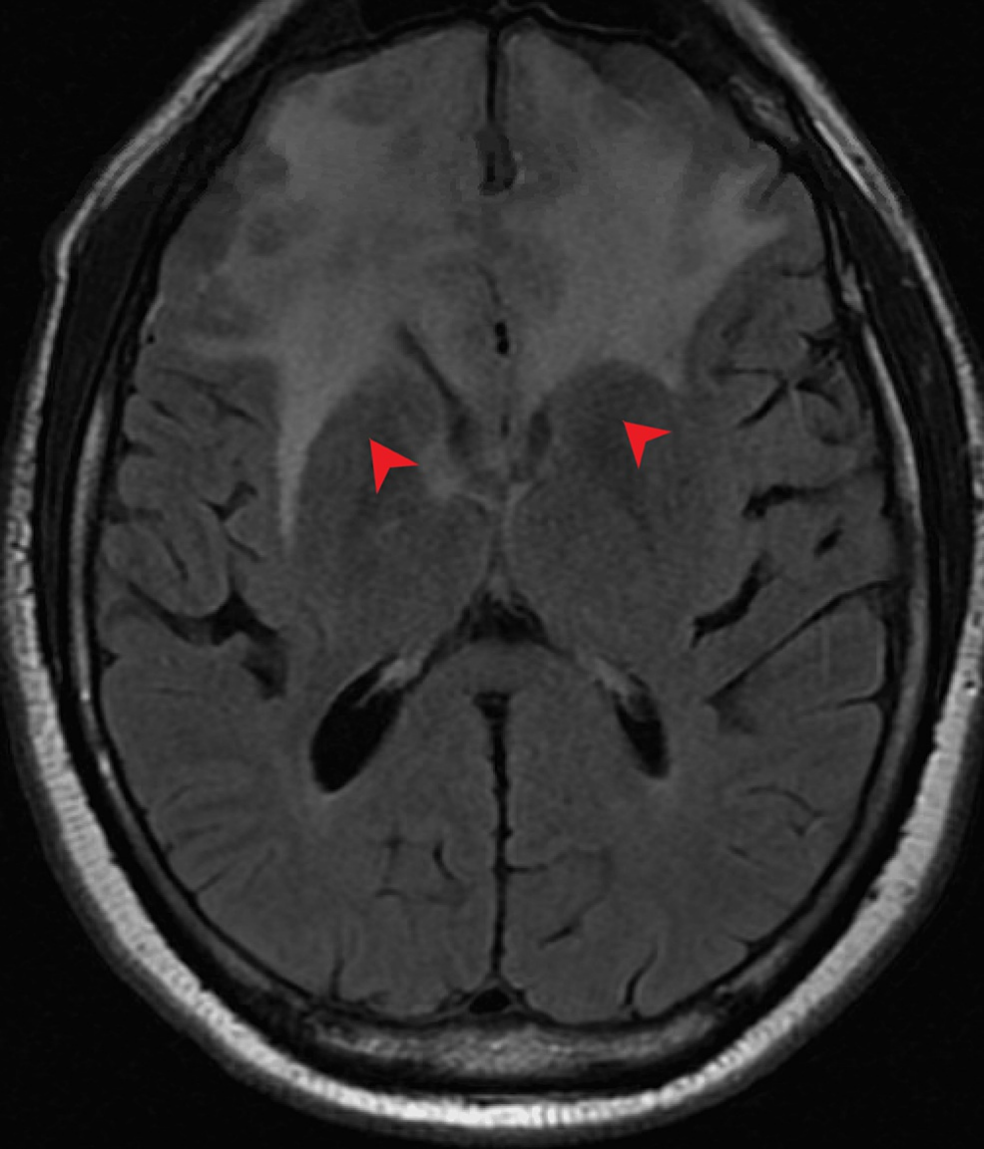
Axial T1-weighted MRI of the brain with contrast revealing extensive nodular leptomeningeal enhancement along the bilateral frontal lobes, suprasellar cistern, right Sylvian fissure, and right basal ganglia with associated extensive vasogenic edema extending to the lateral ventricles (red arrows)

Magnetic resonance imaging (MRI) of the brain with contrast revealed extensive enhancing dural nodularity along bilateral frontal convexities and anterior falx associated with extensive nodular leptomeningeal enhancement along the bilateral frontal lobes, suprasellar cistern, right sylvian fissure, and right basal ganglia with associated extensive bilateral frontal vasogenic edema and mass effect on lateral ventricles (Figures 2, 3).

**Figure 4:**
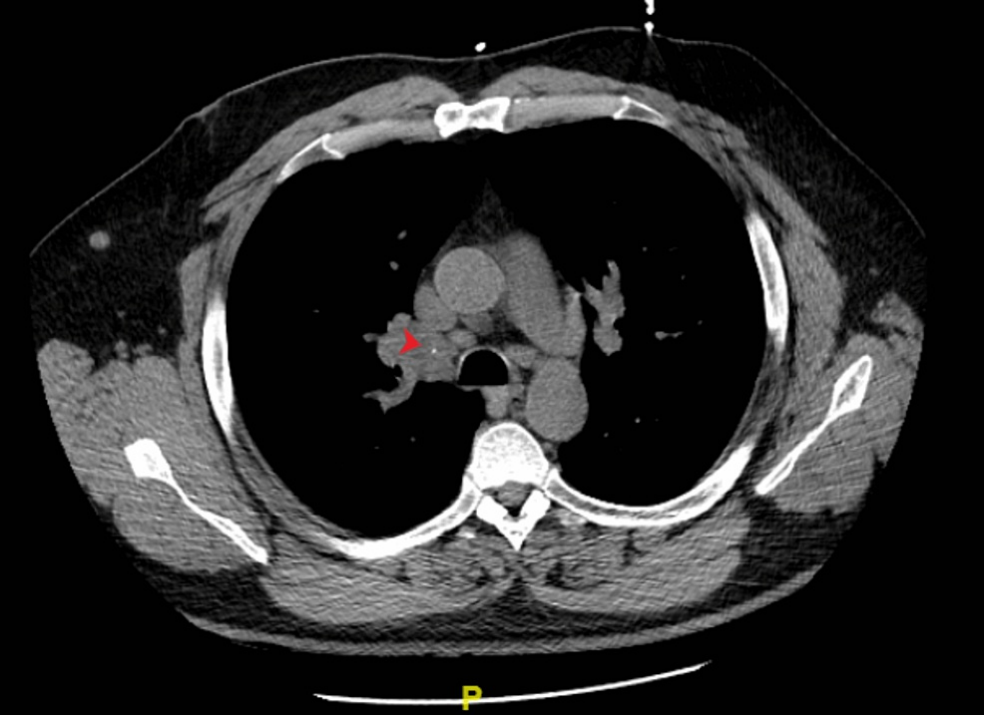
Axial view of chest CT without contrast showing calcified right hilar adenopathy (arrow)

The differentials were narrowed down to central nervous system (CNS) lymphoma versus neurosarcoidosis. Due to concerns of CNS lymphoma, steroids were initially withheld pending cerebrospinal fluid (CSF) analysis. CT of the chest without contrast was done and revealed sub-centimeter mediastinal, bilateral hilar, and right axillary lymphadenopathy (Figure 4). A core needle biopsy of the largest axillary lymph node was performed that returned as benign and reactive. There was an insufficient sample for flow cytometry.

**Figure 5:**
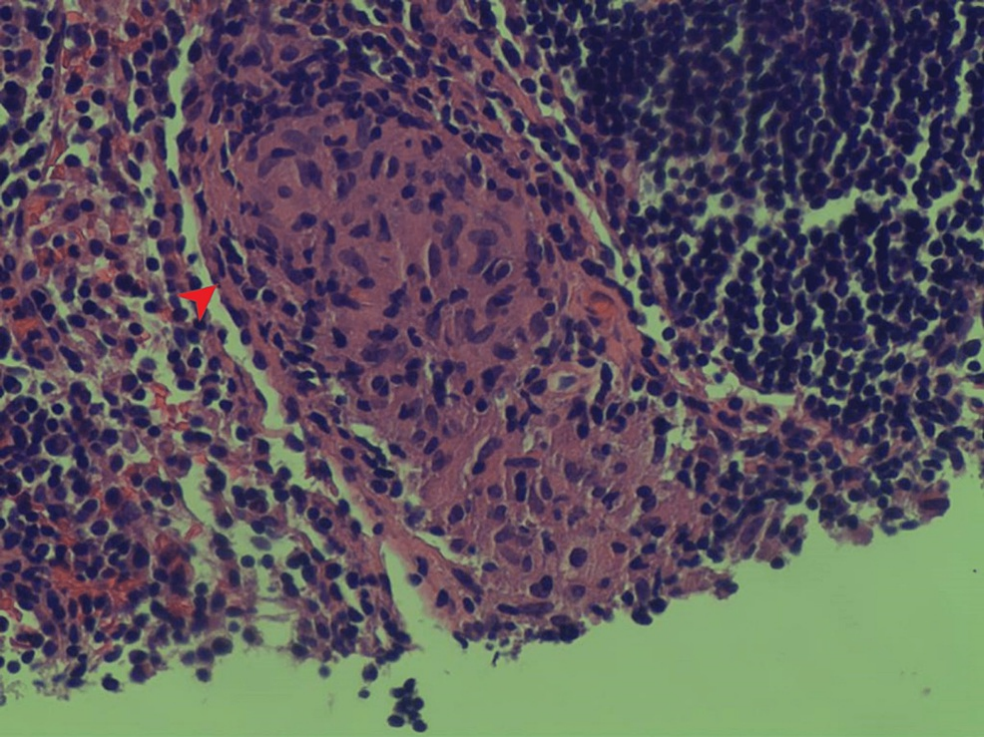
Pathology of the bladder mass showing noncaseating granulomas (arrow)

**Figure 6:**
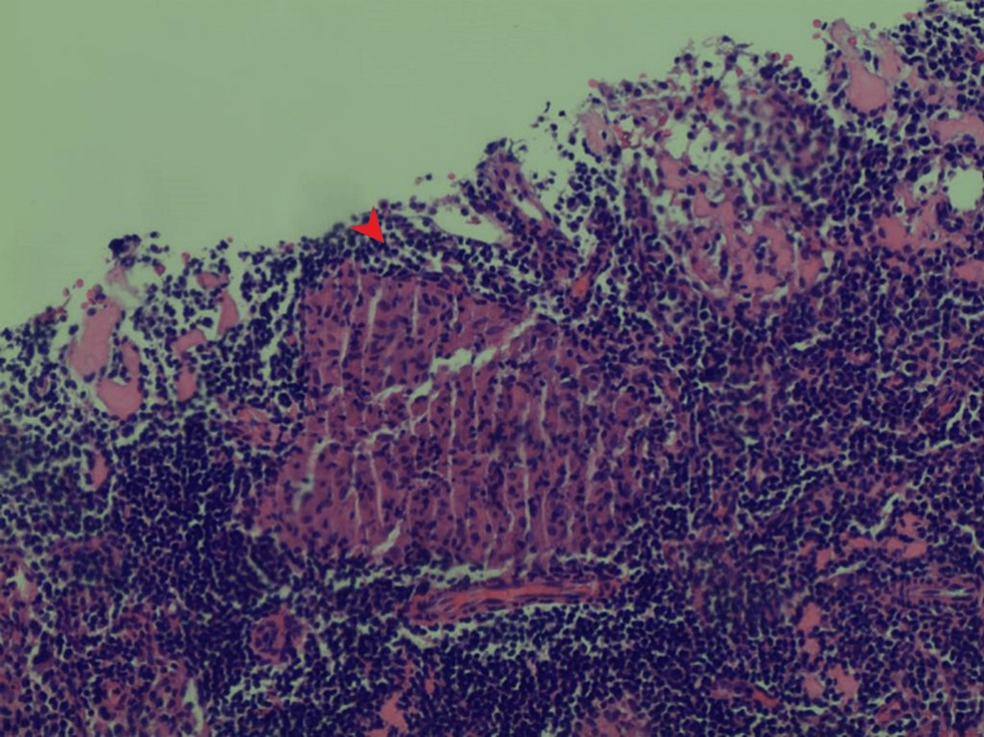
Pathology of the bladder mass showing noncaseating granulomas (arrow)

CT of the abdomen and pelvis with contrast was ordered for further assessment of lymphadenopathy that discovered a 2.6-cm bladder wall tumor concerning for neoplasm. There was also bilateral iliac chain and retroperitoneal adenopathy suspicious for metastatic disease. The patient underwent transurethral resection of the bladder tumor. The pathology revealed noncaseating granulomas (Figures 5, 6).

On hospital day 7, the patient developed new-onset left-sided weakness including reduced grip strength and left-sided facial droop. Brain imaging revealed persistent vasogenic edema. After a discussion with all the specialists, the decision was made to initiate steroids prior to CSF analysis. Acid-fast bacilli (AFB) stains from the axillary lymph node as well as the bladder tumor returned negative. At this time, a lumbar puncture was performed that was negative for infection or malignancy. The CSF angiotensin-converting enzyme (ACE) levels were within normal limits (Tables 1, 2).

**Table 1:** Infectious workup PCR: polymerase chain reaction; Ab: antibody; Ag: antigen; EBV: Ebstein-Barr virus; CMV: cytomegalovirus; HIV: human immunodeficiency virus

Laboratory test	Result	Reference values
Treponemal-specific enzyme immunoassay	<0.2	0.0-0.9
*Neisseria gonorrhoeae* DNA amplification	Negative	Negative
*Chlamydia trachomatis* DNA amplification	Negative	Negative
EBV DNA (PCR)	Negative	Negative
CMV DNA qual (PCR)	Negative	Negative
Hepatitis Bs Ag	Positive	Negative
Hepatitis Bs Ab	0 mIU/mL	>12.0 mIU/mL
Hepatitis Bc IgM Ab	Negative	Negative
Hepatitis Be Ag	Positive	Negative
HIV 1 and 2 Ag/Ab	Nonreactive	Nonreactive

**Table 2:** CSF and serum analysis WBC: white blood cell; RBC: red blood cell; CSF: cerebrospinal fluid; ANA: antinuclear antibody *IgG index was 0.4 (normal reference value 0.0-0.7).

CSF analysis	Result		Reference values
Appearance	Clear		Clear
WBC	3		0-10/CMM
RBC	0		0-3/CMM
Neutrophils %	0		0-6%
Lymphocytes %	55		40-80%
Monocytes %	45		15-45%
Glucose	66		40-70 mg/dL
Total protein	62.5		15-45 mg/dL
Angiotensin-converting enzyme	<1.5		0.0-3.1 U/L
IgG*	7.7		0.0-10.3
Serum analysis			
IgG (serum)		2535	603-1613 mg/dL
IgA (serum)		104.1	70-400 mg/dL
IgM (serum)		<25	40-230 mg/dL
IgE (serum)		159	<100 IU/mL
Free kappa light chain, quantitative		208.7	3.3-19.4 mg/L
Free lambda light chain, quantitative		11.2	5.7-26.3 mg/L
ANA screen		Negative	Negative

The patient clinically improved after the initiation of steroids and a repeat MRI scan of the brain showed moderate interval improvement with persistent leptomeningeal enhancement and resolution of the previously present mass effect on frontal horns. The diagnosis of neurosarcoidosis was favored given bilateral hilar adenopathy on the chest CT scan, improvement in imaging findings following steroid administration, and noncaseating granulomas found in the bladder lesion. Neurosurgery and Oncology did not favor brain biopsy at this time. The patient was discharged on dexamethasone with instructions to follow up with Rheumatology as an outpatient.

## Discussion

Up to 70% of patients with neurosarcoidosis present to medical care with their neurological manifestations rather than already having a known systemic diagnosis [1]. As the disease can present in many different ways without biopsy evidence, solitary nervous-system sarcoidosis is difficult to diagnose [2]. The diagnosis is usually one of exclusion as it often masquerades as other disorders, at times creating a lengthy differential and complicated diagnosis [3]. There is no cure and most patients require long-term treatment with corticosteroids.

Neurosarcoidosis exhibits variable degrees of infiltration in the brain resulting in focal or disseminated nodules or plaques affecting particularly the basal meninges. The granulomatous infiltration may extend into the cortex or white matter causing parenchymal lesions [4-8]. This pattern seen in imaging is generally indistinguishable from that seen with tuberculosis or lymphoma with leptomeningeal involvement [5]. Beside parenchymal lesions and meningeal masses, hydrocephalus may also be seen with MRI.

**Figure 7:**
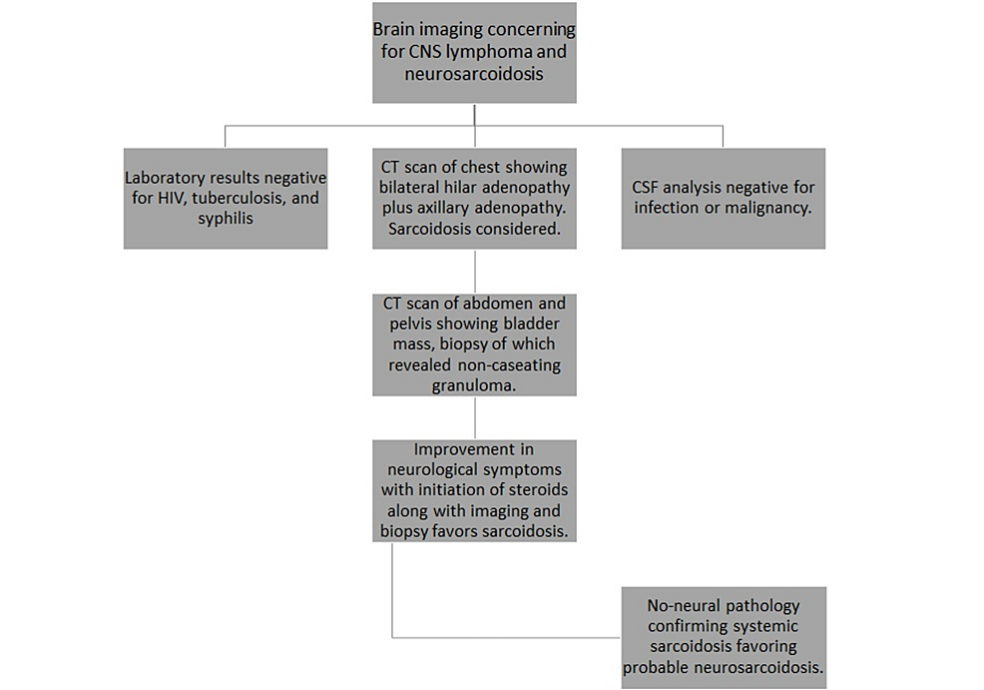
Diagnostic algorithm CNS: central nervous system; CSF: cerebrospinal fluid

In 2018, the Neurosarcoidosis Consortium Consensus Group developed a diagnostic approach for neurosarcoidosis, adapted from 1999, that described possible, probable and definite neurosarcoidosis [9-10]. Alternative diagnoses like infection or malignancy must be ruled out and obtaining a tissue biopsy is recommended. A non-neural pathology confirming systemic sarcoidosis would support a diagnosis of probable neurosarcoidosis, whereas having a neural tissue showing noncaseating granulomas gives a definite diagnosis. If histology is not obtained, having at least two indirect indicators from a gallium scan, chest imaging, and serum ACE is accepted as the confirmation of systemic sarcoidosis. In recent years, F-18 fluorodeoxyglucose (FDG) positron emission tomography (PET) scans have become an important tool to evaluate other sites of involvement in neurosarcoidosis and select more surgically accessible sites for biopsy [11]. If the clinical picture is suggestive of neurosarcoidosis but alternate diagnoses have not been ruled out, then a diagnosis of neurosarcoidosis is thought to be possible (Table 3 and Figure 7).

**Table 3:** Diagnostic criteria

Diagnosis	Criteria
Probable	Non-neural pathology confirming systemic sarcoidosis.
Possible	The clinical picture is suggestive of neurosarcoidosis, but alternate diagnoses have not been ruled out and there is no systemic confirmation of sarcoidosis.
Definitive	Neural tissue showing noncaseating granulomas.

There are several conditions that neurosarcoidosis can mimic. One of them is multiple sclerosis (MS), given the radiologic findings on MRI besides relapsing-remitting course of the disease as well as dissemination of the MRI lesions in space and time. Cognitive and neuropsychiatric presentation in the context of parenchymal brain lesions argues against MS, and certainly, coexisting peripheral nervous system involvement or myopathies are distinctive features of neurosarcoidosis [10]. Additionally, neurosarcoidosis can present with weakness or dysarthria resembling a stroke. Transient ischemic attacks and ischemic stroke due to neurosarcoidosis have been reported [12]. Neurosarcoidosis may coexist with multiple myeloma or be confused with neuropathies secondary to monoclonal proliferation or paraproteinemic neuropathy. The enhancing parenchymal lesions may also be mistaken for neoplasms; however, the lack of central necrosis in histology distinguishes neurosarcoidosis from malignancy. CNS lymphoma is another possible differential diagnosis to keep in mind. There has been a case reported on neurolymphomatosis mimicking neurosarcoidosis. Neurolymphomatosis represents a unique subtype of extranodal lymphoma with localized invasion of cranial or peripheral nerves reported in patients with large B-cell non-Hodgkin's lymphoma [13].

This case is not only a diagnostic challenge but also a therapeutic one due to social constraints. Patient's comorbidities, in addition to homelessness, made it challenging to ensure follow-up with various specialists. Several works of literature have shown sarcoidosis to be a disease that affects those with health disparities [14-15]. According to Sharp et al., “worse dyspnea, lower health-related quality of life, and higher rates of mortality and hospitalization are more common among those who are black, female, or of low socioeconomic status” [14]. Low socioeconomic status is also associated with increased stress, and chronic stress has been shown to impact immune function. A study done by De Vries and Drent showed a direct relationship between stress and sarcoidosis [16]. Also, the economic burden was found to be higher during the first year after diagnosis [17-18]. 

## Conclusions

Neurosarcoidosis poses a diagnostic challenge as it can imitate other neurological diseases, including CNS lymphoma and multiple sclerosis. In addition, the prognosis of neurosarcoidosis varies, with some recovering completely, while others have a chronically progressing, waxing, and waning course. Moreover, it imposes a significant economic burden on the payer, especially in the first year following diagnosis as patients require several specialist visits to manage sarcoidosis-related comorbidities, making it a therapeutic challenge.
